# Case report: Clitoral adenocarcinoma in a mixed-breed female dog

**DOI:** 10.3389/fvets.2023.1264538

**Published:** 2023-09-29

**Authors:** Christina Moss, Nicole Sugai, Rebecca Persons, Brittany Ciepluch, Kevin Lahmers, Julie Cecere

**Affiliations:** ^1^Piedmont Equine Practice, The Plains, VA, United States; ^2^Department of Small Animal Clinical Sciences, Virginia-Maryland College of Veterinary Medicine, Blacksburg, VA, United States; ^3^Department of Biomedical Sciences and Pathobiology, Virginia-Maryland College of Veterinary Medicine, Blacksburg, VA, United States

**Keywords:** clitoris, vulva, prolapse, neoplasia, adenocarcinoma, hypercalcemia, canine

## Abstract

A 9-year-old, spayed female, mixed-breed dog was initially presented for evaluation of chronic dermatitis on the nasal planum, where a clitoral mass was discovered as an incidental finding during the exam. No further investigation of the clitoral mass was undertaken due to other significant dermal lesions and the lack of clinical significance of the mass at the time. However, ~1 month later, the dog was presented to the Emergency Service for bleeding from the vulva. The clitoral mass was found to have prolapsed; the mass was manually reduced back into a position within the vulvar folds and maintained with a purse-string suture. The dog was referred to the Theriogenology Service for further investigation and removal. On follow-up evaluation, the mass was noted to be multi-lobulated, ulcerated, cystic, and involving the clitoris but not the urethra. The urethra was easily catheterized, and no urinary abnormalities were found. No evidence of lymph node metastasis or hypercalcemia was noted prior to surgery. Ultrasonographic evaluation of the anal sacs was normal. The mass was removed, and histopathologic evaluation revealed a primary clitoral adenocarcinoma. On recheck evaluation, after 1 month, no evidence of metastasis or local recurrence was observed. Clitoral adenocarcinoma is a rarely reported neoplasm of the canine genital tract that shares many clinical, histopathological, and immunohistochemical features with canine apocrine gland anal sac adenocarcinoma. This case adds to the available knowledge on the condition, specifically regarding the frequency of complications such as hypercalcemia and metastasis, as previous reports suggest that these are present at least 50% of the time.

## Background

Vaginal and vulvar tumors are uncommon neoplasms in the bitch, representing 3% of all canine neoplasms (1). Specifically, fewer than a dozen cases of canine clitoral adenocarcinoma have been reported in the literature, with the first case being reported in 2010 (2). This condition seems to be similarly rare in humans; the reported incidence of vulvar cancer in women is 4% of all gynecological malignancies with the clitoris being affected in 2–27.7% of cases of vulvar neoplasia (3, 4). Squamous cell carcinoma is the most common malignancy seen in women, representing 95% of all vulvar tumor types (5). The clitoris is composed of fibrous, muscular, and adipose tissue and is covered by a non-keratinized squamous epithelium. There are scattered apocrine glands within the clitoral fibrous and adipose tissue, with the highest density of these glands being at the base of the clitoris and in the clitoral fossa (1). Clitoral adenocarcinomas in the bitch share a variety of clinical, histopathological, and immunohistochemical features with apocrine gland anal sac adenocarcinomas; though in all canine clitoral adenocarcinoma cases previously reported, examined anal glands were normal (1, 2, 6–8). A review of six cases of clitoral adenocarcinoma describes the condition as occurring in spayed females with a median age of 9.5 years (1). Individual case reports of the condition involve animals of similar signalment (2, 6–8). Clinical signs include vulvar licking, perineal swelling and irritation, hemorrhagic vulvar discharge, lower urinary tract signs, and the presence of a visible or prolapsed soft tissue mass. Clitoral masses have also been an incidental finding on physical exam (1, 8). Previous case reports suggest that, in dogs, hypercalcemia of malignancy is present in up to half of the cases of clitoral adenocarcinoma (1, 2, 6, 8). Hypercalcemia of malignancy has most commonly been associated with lymphoproliferative disorders and canine apocrine gland anal sac adenocarcinoma and has not previously been reported to be associated with canine vaginal or vulvar tumors (1, 2). However, the condition has been reported to be associated with canine penile adenocarcinoma (9). If canine clitoral adenocarcinomas are not addressed, metastasis to locoregional lymph nodes has occurred, with histologic confirmation, in 60% of the previously reported cases (1, 2, 7, 8). Additionally, 60% of the previously reported cases of canine clitoral adenocarcinoma resulted in euthanasia for tumor-related reasons (1, 2, 6–8).

## Case presentation

**Figure 1:**
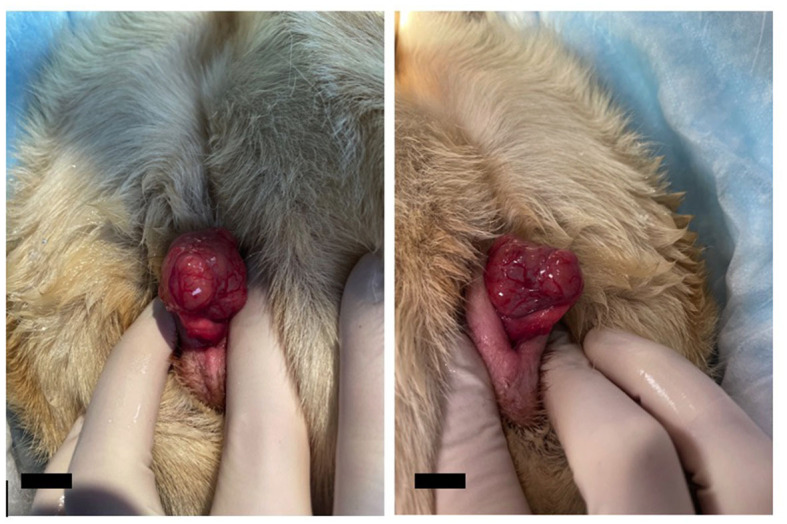
Photos of the clitoral mass during initial examination by the Community Practice service. The mass was noted to be lobulated and associated with the clitoris but was maintained within the vulvar folds at this time. The mass is intentionally prolapsed for better visualization. The scale bar in the bottom left corner of the photograph denotes 1 cm.

A 9-year-old, spayed female, mixed-breed canine presented to the Veterinary Teaching Hospital Community Practice Service at the Virginia-Maryland College of Veterinary Medicine for evaluation of a nose lesion present for 6 months. The dog was initially treated with cephalexin for 14 days, which resolved a secondary bacterial component but did not eradicate the underlying primary skin lesion. The lesion was reported by the owner to have a cyclic appearance, sometimes presenting as inflamed and ulcerated and at other times seeming to regress. No underlying cause had yet been determined. On examination of the dog, vital parameters were within normal limits. Several cutaneous masses on the forelimbs and a vulvar mass (Figure 1) were also noted in addition to the lesion on the nasal planum. Samples of the primary nose lesion and one of the cutaneous masses were obtained for cytologic and histopathologic examination. Analysis of the nose lesion revealed evidence of an autoimmune-mediated disease, likely lupus erythematosus. Analysis of the cutaneous mass from the forelimb revealed a completely excised cutaneous mast cell tumor. Treatment with systemic prednisone was initiated for the autoimmune-mediated process. The vulvar mass was not biopsied at this time due to the potential poor prognosis associated with cutaneous mast cell tumors.

The dog was presented 1 month later to the VTH Emergency Service for evaluation of a bleeding vulvar mass. The owner reported that consistent, mild hemorrhage had been noted from the vulva after the dog had played outside for a period of a few hours. The dog was reported to have recently developed an increased frequency of vulvar licking. On presentation to the Emergency Service, vital parameters were within normal limits, and no peripheral lymphadenomegaly was detected. A large, ulcerated mass was noted protruding from the vulva; the mass was noted to seem larger than it had 1 month before. The perineal area was clipped and aseptically prepared, and then the mass was manually reduced back into the vulva. A purse-string suture was placed to contain the mass within the vulva. Evaluation by the Theriogenology Service was recommended within the next 48–72 h to discuss additional diagnostics and mass removal. No additional medications were prescribed at this appointment.

**Figure 2:**
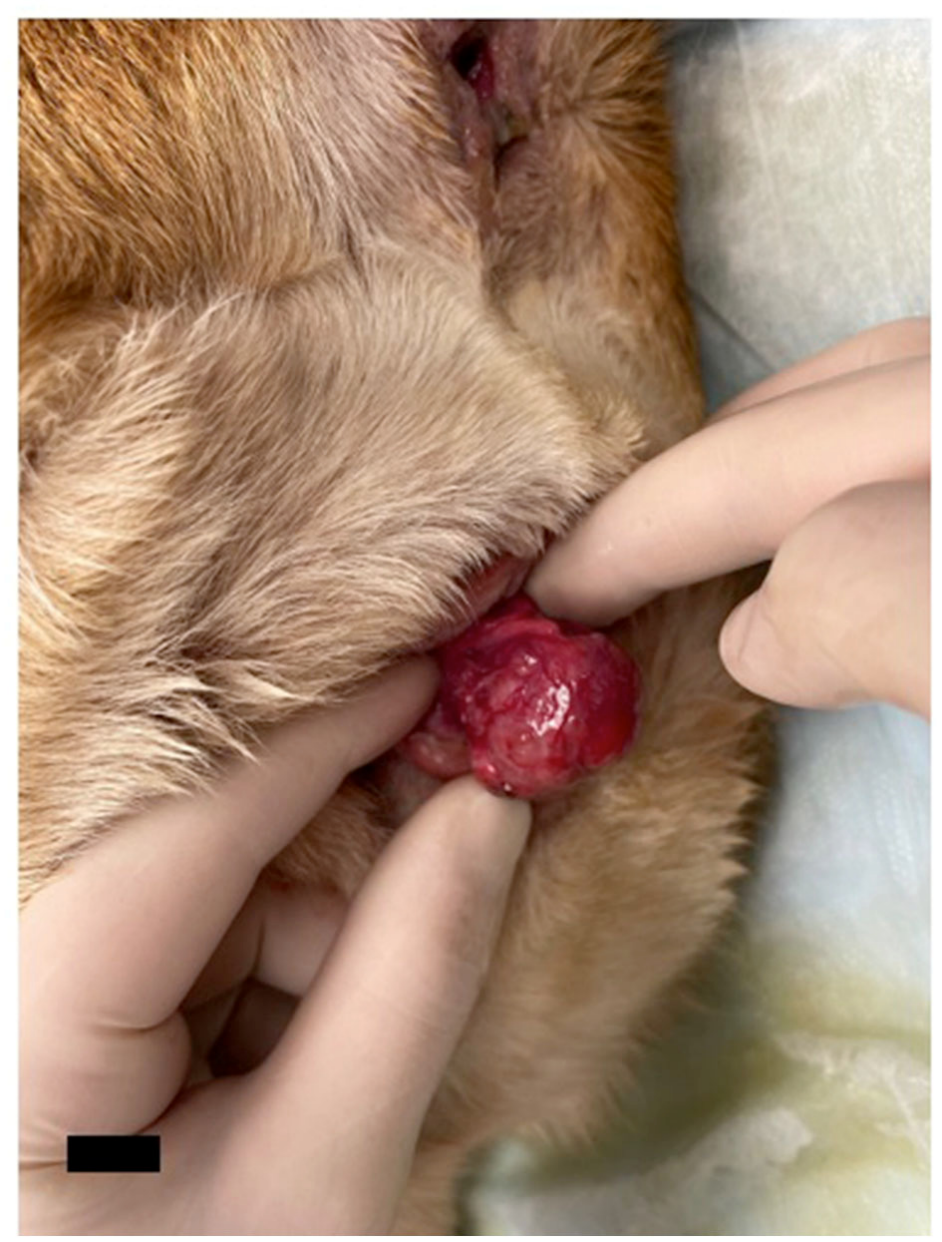
Photo of the clitoral mass four weeks later at presentation to the Theriogenology Service. The mass was noted to be ~2×2 cm, multi-lobulated, and associated with the clitoris but not involving the urethra. It was determined on ultrasound to have cystic areas. The scale bar in the bottom left corner of the photograph denotes 1 cm.

The dog was presented to the Theriogenology Service after 48 h. Her vulva was noted to be red and inflamed with the purse string suture still in place. Pain was noted on palpation of the vulvar region. Hard feces prevented a complete digital rectal exam. A 2x2 cm, multi-lobulated, cystic mass (as demonstrated *via* ultrasonography) was noted on the ventral wall of the vestibule below the clitoris (Figure 2). The distal urethra was grossly normal and could be catheterized. A vaginal stricture was noted cranial to the urethral opening. Serosanguinous vulvar discharge was present associated with the mass. Multiple diagnostics were performed and included complete blood count, serum chemistry, urinalysis, and impression cytology of the mass. A complete blood count revealed evidence of an inflammatory process (mature neutrophilia and monocytosis). Chemistry revealed abnormalities attributed to chronic steroid administration (elevated ALT, ALKP, GGT, and total bilirubin). Creatinine, a byproduct of muscular metabolism, was low, attributable to muscle wasting secondary to chronic glucocorticoid administration (prednisone). No elevation in serum calcium was noted. Urinalysis revealed isosthenuria and proteinuria. Cytology of the clitoral mass revealed numerous parabasal epithelial cells, occasional red blood cells, many neutrophils, and few macrophages. Partial caudal abdominal ultrasound revealed a normal urinary bladder and anal glands, with no evidence of sub-lumbar or medial iliac lymphadenopathy. Due to the inflammation associated with the purse-string suture and concern for lower urinary tract infection prior to surgery, Clavamox was prescribed. Surgical removal of the mass was scheduled for the following week.

The dog returned a week later for surgical removal of the clitoral mass. The patient was placed in sternal recumbency for the surgery with a sterile urinary catheter placed to ensure no urethral involvement during the procedure. Due to the location of a stalk-like attachment, episiotomy was performed on the dorsal commissure of the vulva to allow better access and visualization of the area. Using careful blunt dissection and electrocautery to control hemostasis, the surgeon was able to remove the mass entirely with narrow margins of grossly normal tissue at the level of the stalk attachment. The excision site and episiotomy site were closed in a routine fashion. The entire mass was submitted for histopathology.

**Figure 3:**
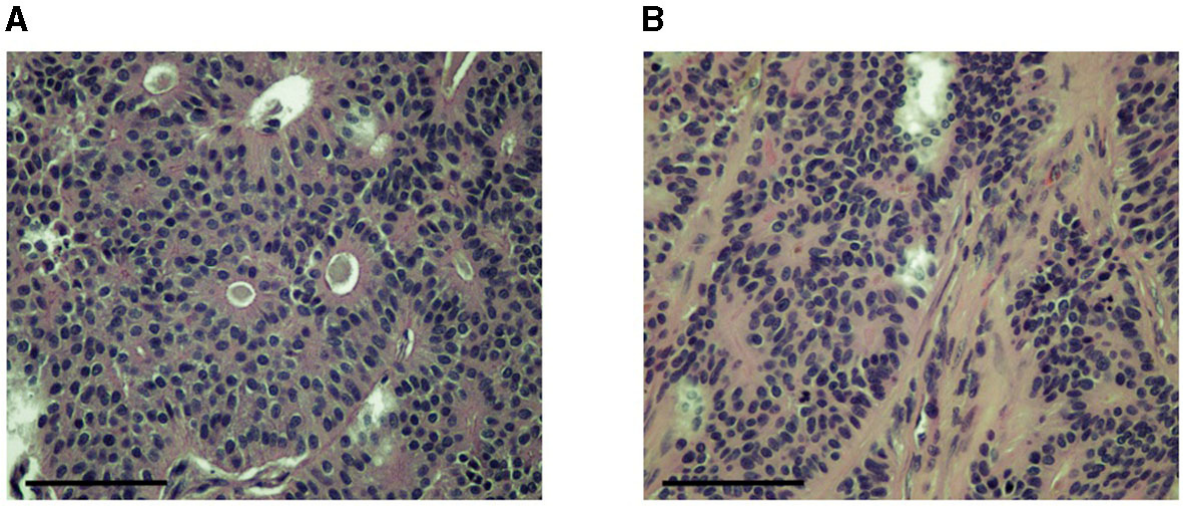
Images of slides made for histopathologic analysis of the tumor showing epithelial cell groups forming characteristic rosette patterns **(A)** supported by a moderate amount of fibrous stroma **(B)**. Images were obtained at 40x. Slides were stained with H&E. The scale bar in the bottom left corner of the photograph denotes 100 um.

Histopathology of the mass revealed an unencapsulated, infiltrative, nodular, densely cellular mass. The mass was composed of cells that formed sheets, tubules, and rosettes supported by a moderate amount of fibrous stroma (Figure 3). Cells were medium-sized and cuboidal with a moderate amount of eosinophilic cytoplasm. Nuclei were medium-sized and round with clumped chromatin. There were 15 mitotic figures per ten 400x fields (1.5 mitotic figures per high power field). Small areas of necrosis were noted scattered throughout the mass. The overlying epithelium was noted to be multifocally ulcerated. Moderate numbers of neutrophils had infiltrated the ulcer beds. The deep margin of the specimen was noted to have a stalk of fibrovascular tissue with 3 mm of normal tissue. A diagnosis of canine clitoral adenocarcinoma was made. Excision appeared to be complete; however, due to the invasive nature of the mass and high mitotic rate, recheck appointments for local recurrence and/or metastasis were recommended. Additional staining procedures (such as for specific cell markers) were not undertaken as part of the pathological analysis of this case.

**Figure 4:**
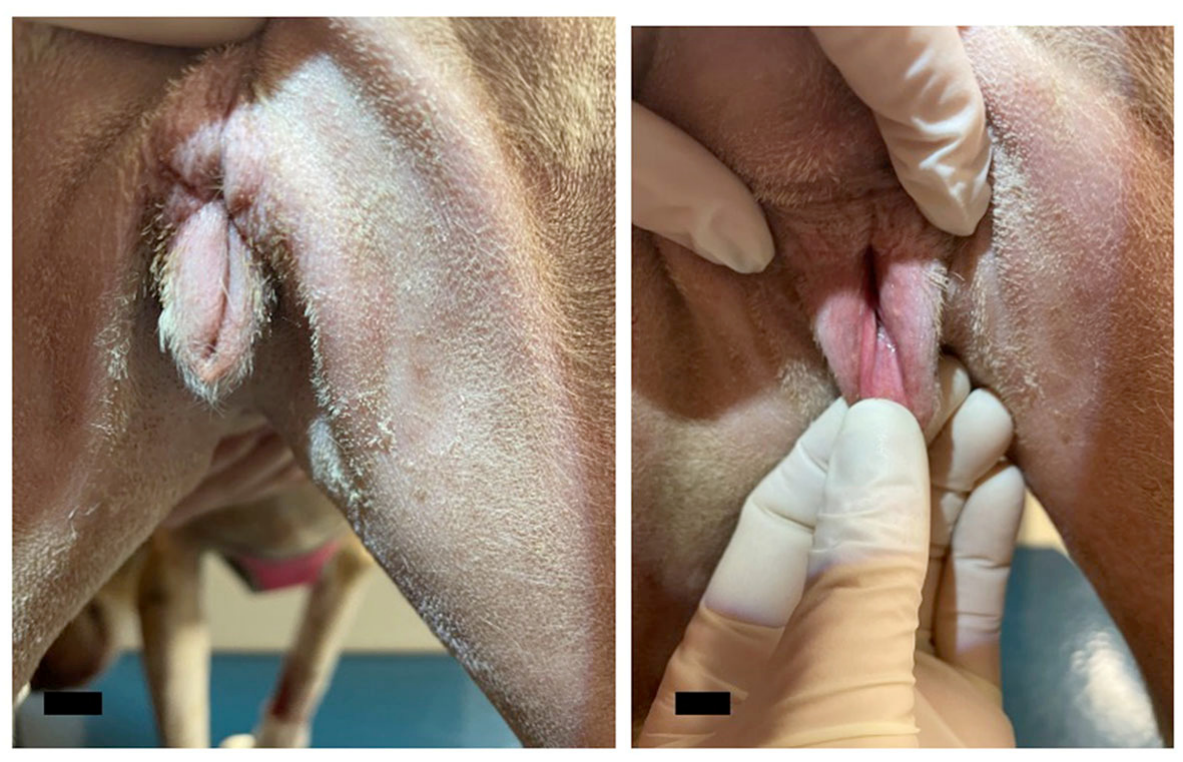
Photographs from the post-operative recheck exam. The post-operative appearance of the vulva was deemed satisfactory with no discharge, dehiscence, or signs of inflammation present. The scale bar in the bottom left corner of the photograph denotes 1 cm.

The dog returned to the Community Practice Service 1 month later for a recheck examination. The surgical site appeared to be healing well (Figure 4), and no enlarged peripheral lymph nodes were detected. Thoracic radiographs were taken and revealed no evidence of pulmonary or intrathoracic lymph node metastasis. Abdominal ultrasound revealed no evidence of metastasis to the intra-abdominal lymph nodes, liver, spleen, or other gastrointestinal structures. Suspected steroid-induced hepatomegaly was noted. Sedated vaginal exam revealed no evidence of local recurrence of the mass. At this time, no evidence of local recurrence or metastasis was evident; the dog was discharged with instructions to return every 6 months for recheck examinations. To date, the patient is doing well, and no evidence of recurrence has been found.

The dog returned after 5 months for constipation and was found to have developed a rectal mass. The mass was shelled out of the rectal mucosa and submitted for histopathology, which revealed it to be a benign leiomyoma.

## Discussion

Vulvar carcinoma has been reported in humans, horses, and bovids, among other species, although these cases are generally of squamous cell carcinoma and may or may not involve the clitoris (3, 10, 11). Vulvar tumors in bitches are relatively rare, accounting for ~3% of all canine neoplastic diseases (12, 13). Tumors most commonly involve the vestibular and vaginal walls and less commonly involve the clitoris (12, 13). The first case of canine clitoral adenocarcinoma was reported in 2010; since that time, only nine additional cases have been described prior to this case (1, 2, 6–8). Our case involved a 9-year-old female, which is consistent with the median age of 9.5 years for the previously reported cases (1). Additionally, the dog in this case report was spayed. Malignant canine vaginal and vulvar tumors have been reported to be more common in spayed vs. intact female dogs (12, 13). Verin et al. speculated on a possible protective effect of sexual hormones in intact bitches, as has been suspected in intact male dogs with the development of prostatic cancer, though no definitive association has yet been demonstrated (1).

Canine clitoral adenocarcinoma shares many similarities with canine apocrine gland anal sac adenocarcinoma. The earliest report of the condition posited that affected individuals may have a displaced embryonic nest of cells (choristoma), leading to a similar lineage of cells in the clitoris as in the anal sacs and thus the ability to develop adenocarcinoma of the clitoris (2). The normal canine clitoris has been shown histologically to contain apocrine glands within the fibrous and adipose tissue (1). The description of additional cases of canine clitoral adenocarcinoma, all without any detectable changes in the anal sacs, has led to the conclusion that the apocrine glands of the clitoris are the true primary source of neoplasia. Though the tumors originate from the clitoris and not the anal sacs, both clitoral and anal sac adenocarcinoma have been associated with hypercalcemia of malignancy, locoregional lymph node metastasis, similar histologic features, and immunostaining for markers such as E-cadherin (2).

Though it was not observed in our case, hypercalcemia has been associated with 50% of the previously reported cases of clitoral adenocarcinoma and, in some cases, may have been the complication leading to the decision for euthanasia (1, 2, 6, 8). Hypercalcemia is most commonly due to a paraneoplastic syndrome in dogs (14). The most common tumor types associated with this paraneoplastic syndrome are lymphoma and apocrine gland anal sac adenocarcinoma, though the condition has also been reported with thyroid carcinoma, multiple myeloma, and mammary carcinoma, among others (14). Hypercalcemia results from tumor secretion of parathyroid hormone-related protein, which was first purified from a human patient with squamous cell carcinoma of the lung in 1987 (15). Mellanby et al. identified high concentrations of the protein in a majority of dogs with hypercalcemia and underlying malignancies, but not in normocalcemic dogs, suggesting that the protein is responsible for aberrant calcium levels (16). Parathyroid hormone-related protein, like the protein it is named for, stimulates bone and renal tubular resorption of calcium, leading to increased serum calcium levels. Prolonged hypercalcemia may lead to anorexia, episodic weakness, polyuria, polydipsia, and renal failure (2, 9). Hypercalcemia has been reported to resolve in cases where the primary clitoral tumor was removed; however, the condition may recur with metastasis to secondary sites (2, 6).

Local lymph node metastasis was confirmed histologically in 60% of the previous case reports of canine clitoral adenocarcinoma. Reported sites of lymph node metastasis include the inguinal lymph nodes, the iliac lymph nodes, and the hypogastric lymph nodes (1, 2, 7, 8). Lymph node metastasis may be detected from a few weeks to many months following primary tumor removal; however, detection of this generally leads to the decision for euthanasia due to poor prognosis (2, 6). In addition to local lymph node metastasis, clitoral adenocarcinomas may show initial invasiveness and local recurrence. In our case, the urethra was easily catheterized and had not been invaded by the primary tumor; however, in one of the previous cases, the involvement of the urethra led to the decision for euthanasia (8). While lymph node metastasis has not been found to be a significant negative prognostic indicator for anal sac adenocarcinomas, metastasis to the lungs is associated with a shorter median survival time, even with treatment (17). At the time of the writing of this study, our case had not shown any evidence of metastasis to lymph nodes or other organs and had no evidence of local recurrence of the tumor.

The histological description of the clitoral adenocarcinoma removed from our case is similar to what has been described in previous cases. These tumors are generally multi-lobulated or nodular and moderately to well demarcated. They can be unencapsulated, partially encapsulated, or fully encapsulated. They often efface the normal architecture of the clitoris (1, 2, 6–8). There are three distinct patterns of epithelial cells observed—tubular, solid, and rosette—and epithelial cell nests are traversed by a fibrovascular stroma (1). Mitotic figures range from 1 to 5 per high power field. The epithelial cell patterns in clitoral adenocarcinomas are also observed in anal sac adenocarcinomas (18). Tumor necrosis was reported on histopathology of a clitoral adenocarcinoma that resulted in euthanasia of the case (8). Interestingly, histopathologic evidence of necrosis in anal sac adenocarcinomas has been shown to be a negative prognostic indicator, lowering median survival time when present by close to 50% (18). Small areas of necrosis were noted on histopathology of the clitoral mass from this case. It remains to be seen if the presence of necrosis carries the same negative prognosis for clitoral adenocarcinomas as it does for anal adenocarcinomas, as the dog in the present case report has not yet had any observable secondary maladies attributable to the presence of the tumor. Cytologic analysis of previous cases of clitoral adenocarcinoma described the presence of large epithelial cells with indistinct borders. Nuclei are round with multiple nucleoli. Anisocytosis and anisokaryosis are present (1, 7, 8). Additionally, previous cases have implemented immunohistochemical analysis for tumor identification and found positive staining for pancytokeratin and E-cadherin, with variable staining of neuroendocrine markers (1, 2, 7). Variable neuroendocrine markers and E-cadherin staining are also observed in apocrine gland anal sac adenocarcinomas (19, 20).

Surgical treatment was pursued in 70% of the previous cases of clitoral adenocarcinoma, with or without adjunctive chemotherapy. Three of the seven dogs undergoing surgery were later euthanized, with the other four either being reported as still alive or having no report of death. Dogs that underwent surgical excision for canine apocrine gland anal sac adenocarcinomas had longer median survival times than those not undergoing surgery as part of their treatment (17). Similar results have been reported by Verin et al. in their review of six cases of clitoral adenocarcinoma (1). In addition to or in place of surgery, other therapies include oral prednisone and carboplatin chemotherapy. While one dog that received adjunct carboplatin treatment as reported by Verin et al. was alive at the time of the writing of that study, Williams et al. found no significant effect of different chemotherapeutic treatments on median survival time in dogs with anal sac adenocarcinoma (1, 17). Our case was already receiving prednisone for an autoimmune-mediated skin condition. Though two previous cases that received prednisone for clitoral carcinoma ended in euthanasia, these are far from significant numbers, so the potential positive effect of steroids for clitoral carcinoma has yet to be elucidated (1, 6).

Metastasis at presentation has been reported in both survivors and non-survivors of canine clitoral adenocarcinoma (1). It has also been reported following initial surgical excision when no lymph node enlargement had been noted on the initial exam (2). While the presence of metastasis does not currently seem to be a significant prognostic factor in determining survival, the presence of hypercalcemia appears to be significant, as 80% of clitoral adenocarcinoma cases in which hypercalcemia was reported ended in euthanasia. Both pulmonary metastasis and the presence of hypercalcemia are significant negative prognostic indicators in cases of anal sac adenocarcinomas. Thus far, few case reports of canine clitoral adenocarcinoma have been published; our ability to give a prognosis for survival will improve with more data relating case outcomes to treatment strategies and secondary issues such as hypercalcemia and metastasis.

## Concluding remarks

Canine clitoral adenocarcinoma is a rarely reported condition with outcomes ranging from no evidence of recurrence or metastasis, as observed in this case, to euthanasia secondary to tumor-related issues. Additional case reports are needed to better characterize the prognosis and incidence of complications associated with this condition so that owners may be well informed when considering treatment options. This case report contributes to the advancement of our knowledge concerning this rare canine neoplasm.

## Data availability statement

The original contributions presented in the study are included in the article/supplementary material, further inquiries can be directed to the corresponding author.

## Ethics statement

The animal studies were approved by Virginia-Maryland College of Veterinary Medicine. The studies were conducted in accordance with the local legislation and institutional requirements. Written informed consent was obtained from the owners for the participation of their animals in this study.

## Author contributions

CM: Writing—original draft. NS: Writing—review and editing. RP: Writing—review and editing. BC: Writing—review and editing. KL: Writing—review and editing. JC: Supervision, Writing—review and editing.

## References

[1] Verin R Cian F Stewart J Binanti D MacNeill AL Piviani M. Canine clitoral carcinoma: a clinical, cytologic, histopathologic, immunohistochemical, and ultrastructural study. Vet Path. (2018) 55:501–9. 10.1177/0300985818759772 29444629

[2] Neihaus SA Winter JE Goring RL Kennedy FA Kiupel M. Primary clitoral adenocarcinoma with secondary hypercalcemia of malignancy in a dog. J Am Anim Hosp Assoc. (2010) 46:193–6. 10.5326/0460193 20439943

[3] Olawaiye AB Cuello MA Rogers LJ. Cancer of the vulva: 2021 update. Int J Gynecol Obstet. (2021). 155:7–18. 10.1002/ijgo.13881 34669204 PMC9298362

[4] Al-Shebaily MM Qureshi VF. Malignancies in clitoris A review of literature on etiology, diagnosis, pathology and treatment strategies. Int J Cancer Res. (2008) 4:110–26. 10.3923/ijcr.2008.110.126

[5] Weinberg D Gomez-Martinez RA. Vulvar cancer epidemiology, clinical presentation, and management options. Int J Womens Health. (2015) 7:305–13. 10.2147/IJWH.S68979 25848321 PMC4374790

[6] Mitchell KE Burgess DM Carrigan MJ. Clitoral adenocarcinoma and hypercalcemia in a dog. Aust Vet Pract. (2012) 42:279–82.

[7] Rout ED Hoon-Hanks LL Gustafson TL Ehrhart EJ MacNeill AL. What is your diagnosis? Clitoral mass in a dog. Vet Clin Pathol. (2016) 45:197–8. 10.1111/vcp.12322 26774097

[8] Chien RC Ritchey JW. Pathology in practice. J Am Vet Assoc. (2021) 259:1–3. 10.2460/javma.256.9.995 32301655

[9] Furtado ARR Parrinello L Merlo M Di Bella A. Primary penile adenocarcinoma with concurrent hypercalcaemia of malignancy in a dog. J Small Anim Pract. (2015) 56:289–92. 10.1111/jsap.12285 25370307

[10] Smith MA Levine DG Getman LM Parente EJ Engiles JB. Vulvar squamous cell carcinoma in situ with viral papillomas in an aged Quarter Horse mare. Equine Vet Ed. (2009) 21:11–6. 10.2746/095777309X390551

[11] Yeruham I Perl S Orgad U Yakobson B. Tumors of the vulva and vagina in cattle - a 10-year survey. Vet J. (1999) 158:237–9. 10.1053/tvjl.1999.0390 10558846

[12] Brodey RS Roszel JF. Neoplasms of the canine uterus, vagina, and vulva a clinicopathologic survey of 90 cases. J Am Vet Med Assoc. (1967) 151:1294–307. 5624188

[13] Thacher C Bradley RL. Vulvar and vaginal tumors in the dog a retrospective study. J Am Vet Med Assoc. (1983) 183:690–2. 6629979

[14] Bergman PJ. Paraneoplastic hypercalcemia. Top Companion Anim Med. (2012) 27:156–8. 10.1053/j.tcam.2012.09.003 23415382

[15] Lai NK Martinez D. Physiological roles of parathyroid hormone-related protein. Acta Biomed. (2019) 90:510–6. 10.23750/abm.v90i4.7715 31910177 PMC7233781

[16] Mellanby RJ Craig R Evans H Herrtage ME. Plasma concentrations of parathyroid hormone-related protein in dogs with potential disorders of calcium metabolism. Vet Rec. (2006) 159:833–8. 17172477

[17] Williams LE Gliatto JM Dodge RK Johnson JL Gamblin RM Thamm DH. Carcinoma of the apocrine glands of the anal sac in dogs. 113 cases (1985-1995). J Am Vet Med Assoc. (2003) 223:825–31. 10.2460/javma.2003.223.825 14507100

[18] Wong H Byrne S Rasotto R Drees R Taylor A Priestnall SL. A retrospective study of clinical and histopathological features of 81 cases of canine apocrine gland adenocarcinoma of the anal sac *independent clinical and histopathological risk factors associated with outcome*. Animals. (2021) 11:3327. 10.3390/ani11113327 34828058 PMC8614406

[19] Suzuki K Morita R Hojo Y Nomura K Shibutani M Mitsumori K. Immunohistochemical characterization of neuroendocrine differentiation of canine anal sac glandular tumours. J Comp Pathol. (2013) 149:199–207. 10.1016/j.jcpa.2013.01.013 23582973

[20] Polton GA Brearley MJ Green LM Scase TJ. Expression of E-cadherin in canine anal sac gland carcinoma and its association with survival. Vet Comp Oncol. (2007) 5:232–8. 10.1111/j.1476-5829.2007.00131.x 19754781

